# Loss of two-pore channel 2 function in melanoma-derived tumours reduces tumour growth in vivo but greatly increases tumour-related toxicity in the organism

**DOI:** 10.1186/s12935-023-03148-6

**Published:** 2023-12-16

**Authors:** Ali Hanbashi, Moureq Alotaibi, Homood M. As Sobeai, Lama Binobaid, Khalid Alhazzani, Xuhui Jin, Faroq Kamli, Ali Alhoshani, John Parrington

**Affiliations:** 1 Department of Pharmacology, University of Oxford, Mansfield Road, Oxford, OX1 3QT UK; 2 Dept. of Pharmacology and Toxicology, College of Pharmacy, King Saud University, Kingdom of Saudi Arabia, P.O. Box 2457, Riyadh, 11454 Saudi Arabia

**Keywords:** Melanoma, Two-pore channel 2, Real-time cell analysis, B16, CHL-1, Tumourigenesis

## Abstract

**Background:**

Melanoma, a severe form of skin cancer, poses significant health risks due to its aggressive nature and potential for metastasis. The role of two-pore channel 2 (TPC2) in the development and progression of melanoma remains poorly understood. This study aims to investigate the impact of TPC2 knockout (KO) on melanoma-derived tumors, focusing on tumour growth and related toxicity in the organism.

**Methods:**

The study utilized CHL-1 and B16 melanoma cell lines with TPC2 KO to assess the changes in proliferation dynamics. Methods included real-time monitoring of cell proliferation using the xCELLigence system, in vivo tumour growth assays in mice, histopathological analyses, inflammation marker assessment, and quantitative PCR (qPCR) for gene expression analysis

**Results:**

TPC2 KO was found to significantly alter the proliferation dynamics of CHL-1 and B16 melanoma cells. The in vivo studies demonstrated reduced tumor growth in TPC2 KO cell-derived tumors. However, a notable increase in tumor-related toxicity in affected organs, such as the liver and spleen, was observed, indicating a complex role of TPC2 in melanoma pathology.

**Conclusions:**

The loss of TPC2 function in melanoma cells leads to reduced tumour growth but exacerbates tumour-related toxicity in the organism. These findings highlight the dual role of TPC2 in melanoma progression and its potential as a therapeutic target. Further research is needed to fully understand the mechanisms underlying these effects and to explore TPC2 as a treatment target in melanoma.

**Graphical Abstract:**

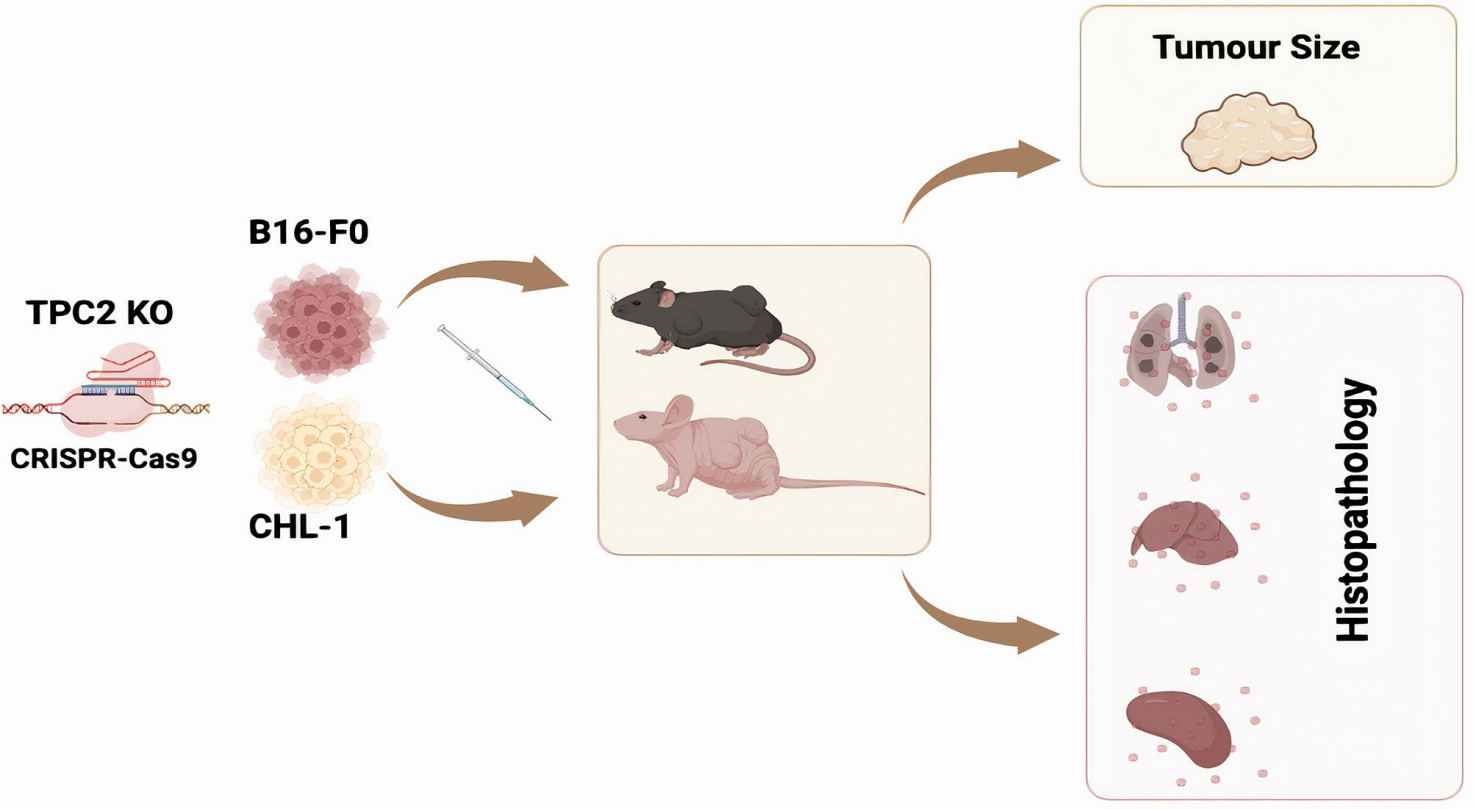

## **Introduction**

Melanoma originates from the tumoural transformation of melanocytes in the skin [1]. The depth of invasion and metastasis of the lymph nodes are very important prognostic features for melanoma classification. Typically, this type of cancer is associated with a very heterogeneous tumour characterised by a high mutational burden (BRAF, NRAS, PTEN, TP53, and CDKN2A are the most frequently mutated genes), and because of this, it is considered a particularly aggressive type of cancer. Therefore, a greater understanding of the molecular and cellular mechanisms underlying melanoma tumourigenesis and metastasis and the identification of new therapeutic targets are important goals for melanoma research. Two-pore channels (TPCs) constitute a pivotal subclass within the extensive voltage-gated cation-channel superfamily, integral to diverse physiological processes. In humans and other primates, this family is delimited to a mere two isoforms, namely: TPC1 and TPC2 [2]. Particularly, TPC2 has been recognized for its selective expression in distinct organelles such as the lysosomes, late endosomes, and melanosomes. This specificity in cellular localization insinuates a potential bespoke role for TPC2 in intricate processes of cellular trafficking or signalling associated with these subcellular compartments. However, despite the burgeoning interest in TPC2, its exact *modus operandi* remains an enigma. Central to the ongoing discourse is the ambiguity surrounding the biophysical attributes intrinsic to TPC2. A bone of contention within the scientific community lies in discerning whether its activation is predominantly mediated by the small molecule nicotinic acid adenine dinucleotide phosphate (NAADP) or if it is preferentially modulated by the lipid signalling molecule, phosphatidylinositol 3,5-bisphosphate (PI(3,5)P2) [3, 4]. Evidently, elucidating the nuances of TPC2’s regulatory dynamics is imperative for advancing our understanding of its physiological implications.

Recent studies have identified important roles for two-pore channel-2 (TPC2) in melanoma [5–8].

However, TPC2’s effects might be nuanced based on the pigmentation and tumourigenesis stages of the melanoma cells. Netcharoensirisuk et al. observed that TPC2 KO in the pigmented MNT-1 human melanoma cell line decreased the cells’ migratory and invasive properties [7]. On the other hand, when we [5] introduced a TPC2 KO in the non-pigmented CHL-1 melanoma cell line, an enhancement in migratory and invasive behaviour was seen. This highlights the intricate relationship between TPC2’s role and melanoma cell characteristics such as pigmentation, emphasizing the importance of understanding the nuanced behaviours of different melanoma cell lines like CHL-1 and MNT-1. Further studies have shown that these changes are caused by the activation of genes regulated by the yes association protein (YAP) and PDZ-binding motif (TAZ), which are key regulators of tumourigenesis and metastasis. Similar effects were observed following TPC2 knockdown using anti-TPC2 siRNAs, confirming that these effects were specifically caused by the loss of TPC2 function. Expression levels of ORAI1, a component of store-operated Ca^2+^ entry (SOCE), and PKC-ßII, a part of the HIPPO pathway that negatively regulates YAP/TAZ activity, were reduced by TPC2 KO and siRNA knockdown. Based on this, we proposed a cellular mechanism mediated by ORAI1/Ca^2+^/PKC-ßII to explain our findings. We did not see such a mechanism involving YAP/TAZ activation in B16 cells, which are a pigmented melanoma cell line derived from a primary mouse melanoma [5].

In line with the different effects of TPC2 KO in these cell lines, patients with metastatic melanoma showed reduced TPC2 expression compared to those with early-stage tumours [5]. This difference has potential clinical relevance, as it suggests that inhibiting TPC2 in melanoma through anticancer chemotherapy might be a valid strategy in the early stages of tumourigenesis, since loss of TPC2 function seems to decrease the migratory and invasive characteristics of early-stage melanoma cells, but not at later stages when inhibition of TPC2 by drugs might actually enhance the metastatic properties of melanoma cells. However, to further confirm the significance of these findings, it will be important to study other melanoma cells at different stages of tumourigenesis and metastasis to confirm that the apparent difference in the role of TPC2 in melanoma cells is linked to the stage of tumour development. Another potential limitation of existing studies that have examined the role of TPC2 in melanoma is that they have all been performed using a variety of in vitro assays.

However, tumourigenesis and metastasis in humans or other organisms are in vivo processes, and findings obtained using in vitro assays may provide only partial or misleading information regarding the role of TPC2 in melanoma tumourigenesis and metastasis. For this reason, in this study, we aimed to investigate what happens when WT or TPC2 KO B16 or CHL-1 melanoma cells are injected into C57BL/6 or nude mice, respectively.

## Methods and materials

### Cell lines and cell culture

B16-F0 (ATCC CRL-6322) murine melanoma cells and CHL-1 (ATCC) human melanoma cells were cultured in Dulbecco’s modified Eagle’s medium (DMEM; Sigma) supplemented with antibiotics (P/S, Sigma), 10% foetal bovine serum (Gibco), and 2 mmol/L glutamine (Sigma). Cells were grown in tissue culture flasks at 37 °C in a 5% CO_2_ humidified environment. B16 KO and CHL-1 KO cells were generated as described in our previous work (D’Amore et al., 2020) [5].

### Real-time monitoring of cell proliferation by an xCELLigence system

This assay used an xCELLigence real-time cell analysis (RTCA) DP instrument (ACEA Biosciences, San Diego, CA, USA) that was set to 37 °C and 5% CO_2_. Experiments were conducted using modified microelectrodes attached to the bottom of 16-well plates to determine the attachment direction, cell distribution, and proliferation based on impedance. First, 50 µl of cell-free growth medium (10% FBS) was added to each well. The plates were incubated at room temperature for approximately 30 min; following this, the background impedance of each well was measured for 30 min. At that point, the cells were harvested directly from the exponential phase cultures using a standardised detachment procedure with 0.05% trypsin-EDTA and counted using the LUNA device (Logos Biosystem, Seoul, South Korea). The cells were then seeded directly into the wells using 50 µl of the cell suspension (5 × 10^3^ cells per well). The plates were left at room temperature for approximately 30 min to allow for cell attachment and then placed under lock in the RTCA DP device, which was positioned in the incubator; the xCELLigence system automatically monitored the impedance value of each well. These values were expressed as confidence intervals (CI) (Cell Index). The xCELLigence system was used to automatically monitor the Cell Index in each well every 15 min. The cells were monitored in real time for five days.

### In vivo tumour growth assay

Animals were provided by the Experimental Surgery and Animal House of the College of Medicine, King Saud University. Male nude and C57BL/6 mice (*N* = 6 animals in each group) aged six to eight weeks were subcutaneously injected with 1 × 10^6^ CHL-1 and 2 × 10^6^ B16 cells, respectively, into the right flank. Animals were housed in a controlled environment and grouped into B16 WT, B16 TPC2 KO, CHL-1 WT, and CHL-1 TPC2 KO groups. Over the course of the study, the tumour volume and body weight were measured and recorded twice weekly. The calculation of tumour volume was performed using the subsequent formula: Volume = 0.52 × (width)2 × (length) [9]. The study duration was 28 days for mice injected with B16 and 24 days for animals injected with CHL-1. The animals were handled by the Guide for the Institutional Animal Care and Use Committee (IACUC) by the National Institute of Health (NIH). The experimental protocol (KSU-SE-20-62) was approved by the Research Ethics Committee of King Saud University. The protocol established a humane endpoint for the study’s tumour growth (2,000 mm^3^). This aligns with the animal care and use guidelines established by our ethical committee and various institutions, including the IACUC, and the Association for Assessment and Accreditation of Laboratory Animal Care (AAALAC). In order to conduct a comprehensive comparison analysis of TPC2 knockout (KO) derived tumours and wild-type (WT) derived tumours, it was imperative to permit the tumour volumes to be beyond the established limits outlined in the recommendations.

### Histopathology

The mouse livers, lungs, and spleens were removed at the end of the experiment, cut into small pieces, and fixed in 10% neutral-buffered formalin. Dehydrated samples were embedded in wax and sectioned to a thickness of 5 μm. HER was used to stain the sections, which were then examined and photographed using a microscope (Motic-2000). Sections of the spleen were subjected to area measurements of the lymph nodes using microscope programs at a magnification of 100× for ten fields in each group, and the volume was calculated using the following formula: The volume of a sphere is equal to 4/3)πr^3^. The data were analysed statistically using the ANOVA test, with a P value of 0.05 considered significant. Data are expressed as the standard error of the mean (SEM).

### Assessment of inflammation markers in mice

Serum cytokine and chemokine concentrations were determined using a 48-plex (Eve Technologies, Canada, cat#HD48) multiplex immunoassay, including: sCD40L, epidermal growth factor (EGF), Eotaxin, FGF-2, Flt-3 ligand, Fractalkine, G-CSF, GM-CSF, GROα, IFNα2, IFNγ, IL-1α, IL-1β, IL-1ra, IL-2, IL-3, IL-4, IL-5, IL-6, IL-7, IL-8, IL-9, IL-10, IL-12p40, IL-12p70, IL-13, IL-15, IL-17 A, IL-17E/IL-25, IL-17 F, IL-18, IL-22, IL-27, IP-10, MCP-1, MCP-3, M-CSF, MDC (CCL22), MIG, MIP-1α, MIP-1β, PDGF-AA, PDGF-AB/BB, RANTES, TGFα, TNFα, TNFβ, and VEGF-A. Serum concentrations of a panel of soluble cytokine receptors, including sCD30, sEGFR, sgp130, sIL-1RI, sIL-1RII, sIL-2Rα, sIL-4R, sIL-6R, sRAGE, sTNFRI, sTNFRII, sVEGF-R1, sVEGF-R2, and sVEGF-R3, were measured using a multiplex immunoassay (Millipore MILLIPLEX; cat# HSCRMAG32KPX14).

### Quantitative PCR (qPCR)

RNA was isolated from cell lysates collected from cultivated cells or homogenised tumour tissue using the RNeasy kit (QIAGEN, #75,144) according to the manufacturer’s instructions. Quantitative PCR (qPCR) was performed using the SYBR Select Master Mix (PowerUP, Thermo Fisher Scientific) according to the manufacturer’s instructions. For each reaction, cDNA (20 ng) and forward and reverse primers (10 pmol) were added to 10 µl of 2 × SYBR Select Master Mix with SYBR GreenER Dye. RNase-free water (20 µl) was added to make up the total volume. The reaction mixture was then loaded onto the Applied Biosystems QuantStudio 5 Real-Time PCR System to measure expression relative to a housekeeping gene. The qPCR was performed with primers specific for TGFβ-1 and IL-6 (Invitrogen). Actin was used as an internal control. The Ct method was used to determine relative mRNA expression levels.

## Results

### TPC2 KO altered the proliferation dynamics of CHL-1 and B16 melanoma cells

**Fig. 1:**
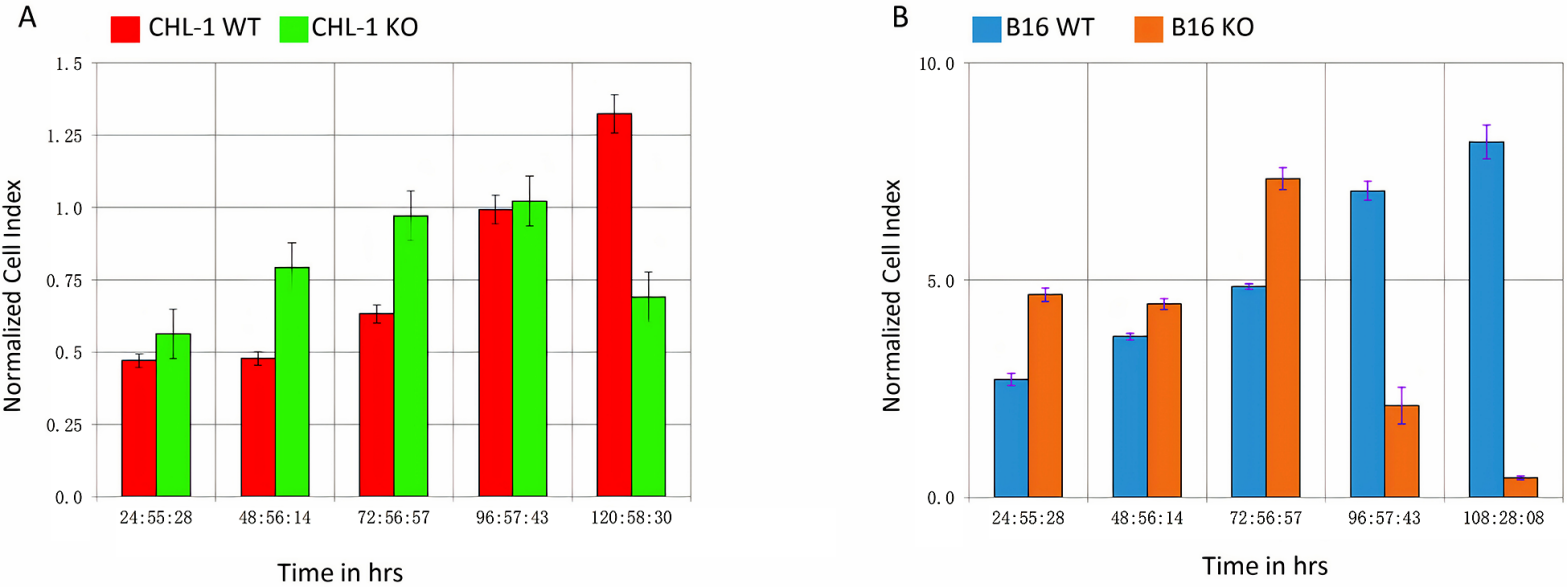
Proliferation dynamics of CHL-1 and B16 (WT and TPC2 KO) cells. **(A)** RTCA proliferation values of CHL-1 WT and TPC2 KO cells. **(B)** RTCA proliferation values of B16 WT and TPC2 KO cells

As a preliminary step towards injecting WT or TPC2 KO B16 or CHL-1 melanoma cells into mice, we studied their cell proliferation properties in vitro using the xCELLigence RTCA system. The RTCA system is a cutting-edge device that enables the real-time monitoring of cell growth via a label-free cell-based assay that monitors impedance fluctuations in culture media. Cell index reflects not only proliferation rate, but also morphological modifications and the quality of cell-electrode interactions. Normalised CI curves (Fig. 1A, B) were generated for all impedance profiles during a 120-h period. They were calculated using the CI, which is a dimensionless relative value that represents the impedance change divided by the background value and thus indicates the total number of cells and the quality of their attachment. The CI fluctuated with time, producing time-dependent impedance profiles during the experiment. The normalised CI values for CHL-1 WT cells were 0.43, 0.47, 0.63, 0.99, and 1.32 for every 24 h, while those for CHL-1 TPC2 KO cells were 0.56, 0.79, 0.97, 1.02, and 0.6 (Fig. 1A). These results indicated that TPC2 KO had an initial positive effect on the initial stages of CHL-1 growth and then further accelerated it. However, after 100 h, CHL-1 TPC2 KO cells displayed a drastic reduction in cell index, whereas WT cells continued to show an increase in this parameter.

A similar RTCA experiment was performed for B16 WT and TPC2 KO cells. The normalised CI values for B16 WT cells were 3.2, 4.4, 5.7, and 8.4 for each of the 24-h periods, while those of B16 TPC2 KO cells were 9.3, 9.6, 15.3, and 4.4 (Fig. 1B). These findings show that TPC2 KO initially enhanced CI values for three days after application, but similar to CHL-1 TPC2 KO cells, the CI rate of B16 KO cell was reduced on the fourth day, while the B16 WT cell rate remained constant.

### TPC2 KO in melanoma cell lines impaired tumour growth

**Fig. 2:**
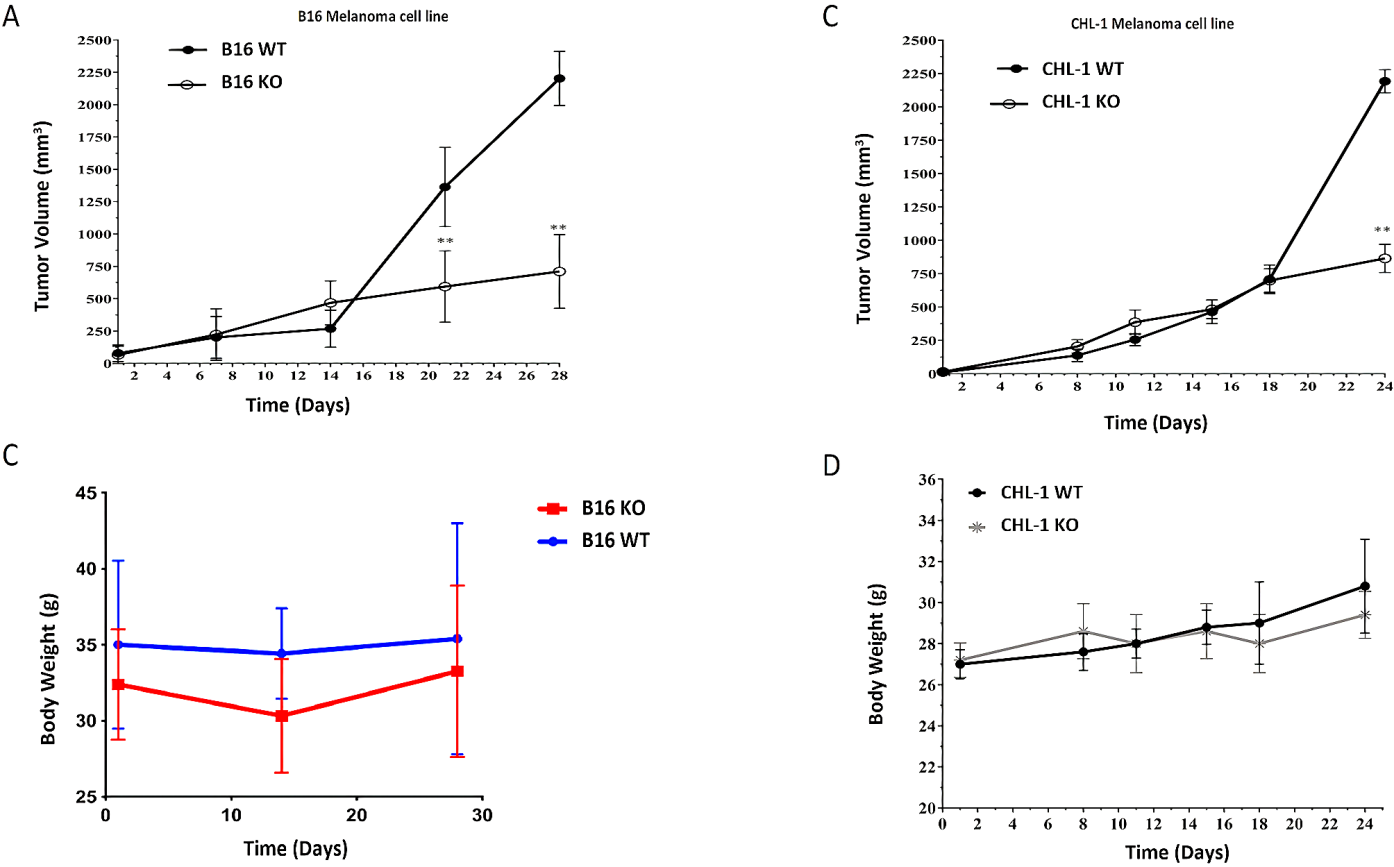
Tumour growth and body weight change in mice subcutaneously injected with either WT or TPC2 KO melanoma cells and monitored in vivo. **(A)** Tumour growth after B16 cell injection. **(B)** Body weight monitoring following B16 cell injection. **(C)** Change in tumour growth after CHL-1 cell injection. **(D)** Body weight monitoring following CHL-1 cell injection. Data was presented as mean ± SD

Despite the numerous benefits of in vitro cancer models, in vivo systems are necessary to evaluate the contributions of the tumour microenvironment, the effect of the host immune system, and processes ranging from angiogenesis to tumourigenesis and metastasis. The subcutaneous injection of cancer cells into mice often results in tumour growth at the injection site. In this study, we injected B16 WT and TPC2 KO cells into C57BL/6 mice and monitored tumour growth and changes in body weight. No significant differences in tumour volume were observed between the two groups of mice in the early post-transplantation days. However, mice injected with WT B16 melanoma cells showed significantly greater tumour growth than mice injected with B16 TPC2 KO cells at 21 days post-injection, and this pattern continued for the remainder of the experiment (Fig. 2A). The TPC2 KO group showed a modest reduction in body weight at the beginning of the experiment; however, this was not statistically significant. Toward the end of the experiment, both groups showed a slight decrease in body weight, consistent with an increased tumour burden. Throughout the experiment, there were no statistically significant differences in body weight between groups (Fig. 2B).

To examine whether TPC2 deficiency in human CHL-1 cells affects tumourigenesis in vivo in mice, nude immunocompromised mice were injected with both WT and TPC2 KO CHL-1 cells. Both cell lines grew slowly at the start of the experiment; however, by 24 days post-injection, the WT group showed significantly greater tumour growth than the TPC2 KO group (Fig. 2C). We also analysed the body weight; neither group showed a statistically significant difference in body weight (Fig. 2D).

### Growth of a TPC2 KO cell-derived tumour led to severe pathological changes in the spleen, liver, and lung

To examine the effect of WT and TPC2 KO cell-derived tumours on the organs of the injected mice, histopathological analysis of different organs was performed at the end of the in vivo experiment. This analysis was performed on organs from animals that were not injected (as negative controls) as well as those injected with either WT or TPC2 KO cells.

**Fig. 3:**
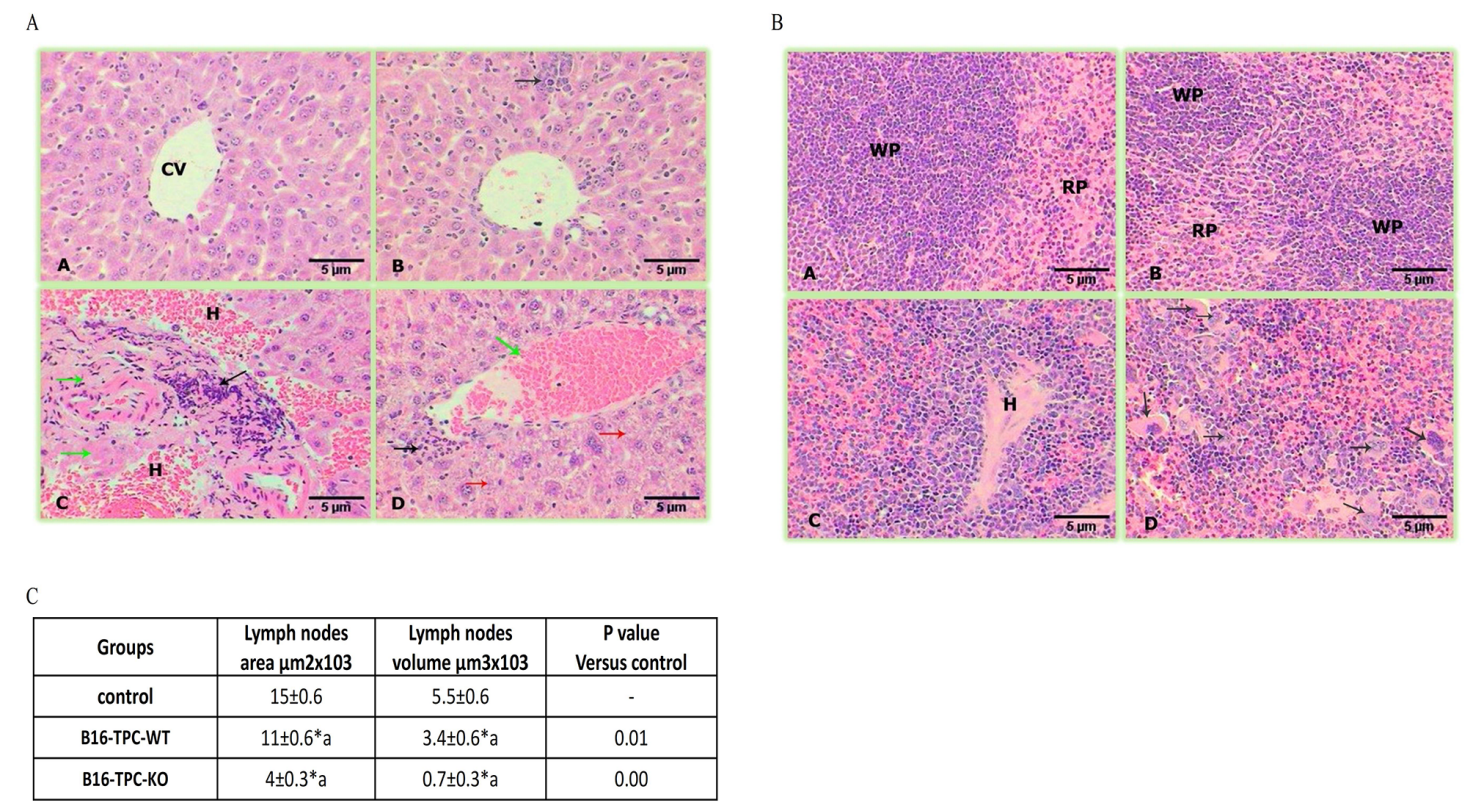
Photomicrographs of liver and spleen tissues. **A)** Liver tissue: **(A)** control group showing normal structure; **(B)** liver cells from B16 WT-injected mice revealing small foci of infiltrative cells (black arrow); **(C)** liver cells from B16 TPC2 KO-injected mice displaying fibrosis (green arrows), aggregations of infiltrative cells (black arrow), and haemorrhage **(H)**; **(D)** liver cells from B16 TPC2 KO-injected mice displaying a congested vein with haemorrhage and oedema (green arrow), infiltrative cells (black arrow), and cytoplasmic degeneration (red arrows). (HE-400X). **B)** Spleen tissue: **(A)** control spleen showing well-defined splenic structure (WP), white pulp (RP), and red pulp; **(B)** spleen tissue from B16 WT-injected mice revealing healthy spleen tissue, (WP) white pulp, and (RP) red pulp; **(C)** spleen tissue from B16 TPC2 KO-injected mice displaying pathological changes with a minimized lymph node centred with hyaline degeneration **(H)**; **(D)** spleen tissue from B16 TPC2 KO-injected mice presenting an ill-defined structure and numerous large macrophages (black arrows). **C)** Table showing a significant decrease in the spleen lymph node area and volume in the B16 WT and B16 TPC2 KO groups versus the control

In spleen tissues, the control mice spleens displayed normal architecture with a well-defined white pulp consisting mainly of lymphocytes and a red pulp that consisted of splenic parenchyma called the cords of Billroth (Fig. 3A-a). The B16 WT-injected group showed a well-defined splenic structure, but with less lymph node area and volume compared to the control group (Fig. 3A-b, C). Remarkably, the third group, B16 TPC2 KO-injected mice, showed major pathological alterations in the spleen represented by hyaline degeneration in the lymph nodes, which in turn appeared minimised and showed a significant decrease in lymph node areas and volumes compared to the control group. Additionally, an ill-defined appearance with white pulp diffused in the red pulp, which was mostly congested, was observed, as was the presence of numerous large macrophages (Fig. 3A-c-d, C).

Control mouse livers showed a normal appearance, consisting of veins surrounded by strands of hepatocytes separated from each other by blood sinusoids with abundant rod-shaped Kupffer cells (Fig. 3B-a). In the second group, the livers of the B16 WT-injected group displayed a relatively healthy hepatic structure but with small foci of inflammatory cells besides the activation of Kupffer cells in the sinusoids (Fig. 3B-b). In contrast, the hepatic structure in the third group, B16 TPC2 KO-injected mice, revealed severe alterations manifested by the incidence of fibrosis surrounded by massive infiltrations that were deposited in wide necrotic areas (Fig. 3B-c). Moreover, congested veins with erythrocytes and oedema with thickened walls appeared in the section in addition to cytoplasmic degeneration of hepatocytes (Fig. 3B-d). Overall, the liver and spleen tissues of mice injected with B16 TPC2 KO cells exhibited far more pathological characteristics than those of mice injected with B16 WT melanoma cells.

**Fig. 4:**
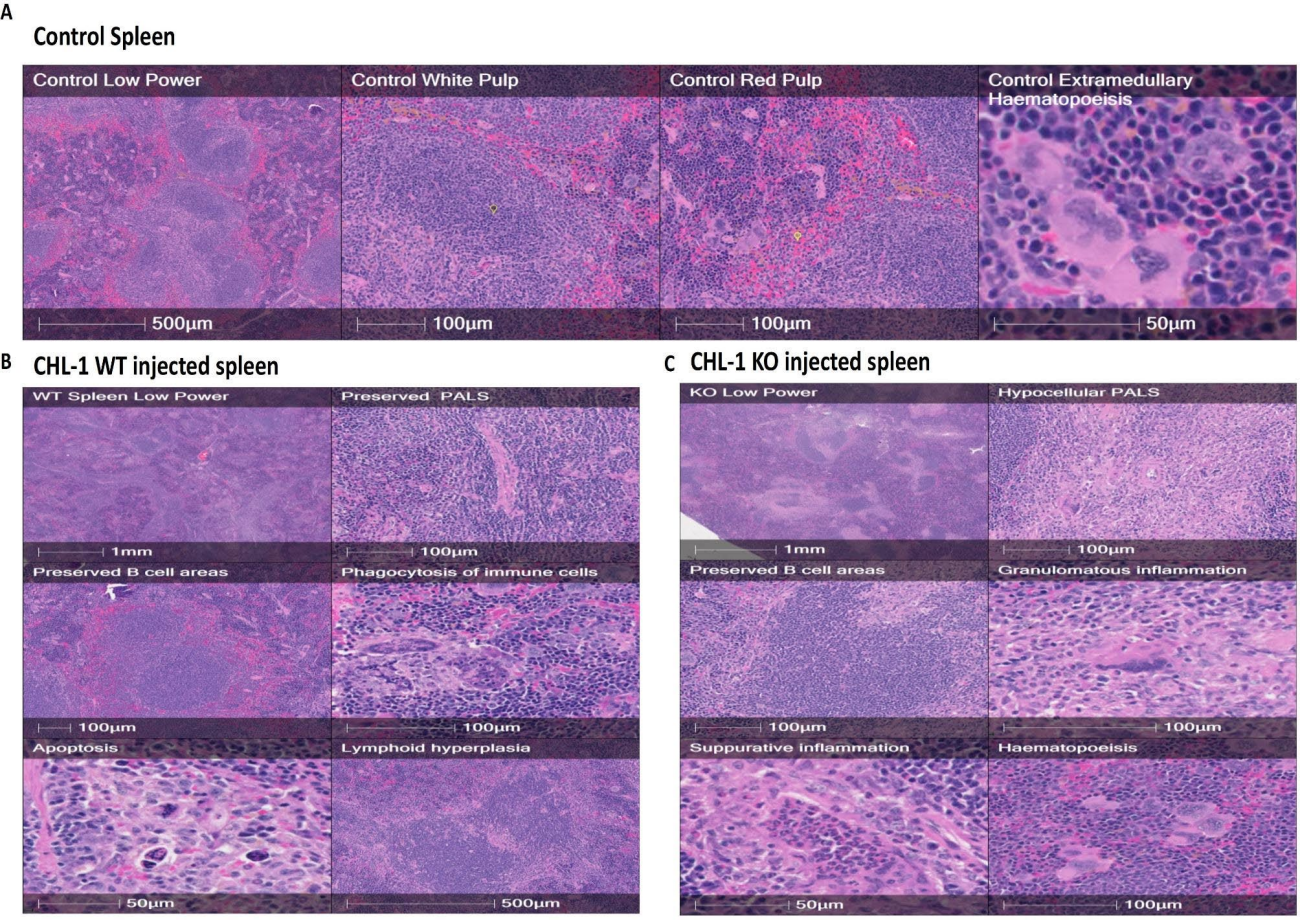
Photomicrographs of spleen tissues 5 weeks after injecting CHL-1 WT or TPC2 KO melanoma cells. **(A)** Control spleen tissue. **(B)** CHL-1 WT-injected mouse spleen tissue. **(C)** CHL-1 TPC2 KO-injected mouse spleen tissue

Histopathological analysis was performed on tissues from mice injected with CHL-1 WT or TPC2 KO melanoma cell lines. This revealed an overall pattern similar to that of B16 cells, but with some notable differences. CHL-1 TPC2 KO-injected mouse spleens showed lymphocyte atrophy in the periarteriolar lymphoid sheath (PALS) (T cells) and macrophage replacement. These spleens had a greater degree of suppurative (neutrophil aggregation) and granulomatous (activated/multinucleated macrophages) inflammation than the spleens of control mice (where this was absent) or WT-injected mice. WT-injected mouse spleens lacked pale patches around the PALS and were instead abundant in lymphocytes, which can be interpreted as lymphoid hyperplasia. Numerous apoptotic cells and widespread phagocytosis of lymphocytes, neutrophils, and apoptotic debris by multinucleated macrophages have been observed. This was more prevalent in the WT-injected mice than in the TPC2 KO-injected mice. Neutrophils from WT-injected mice were more apoptotic than those from TPC2 KO-injected mice. In addition, more macrophages were phagocytosed (Fig. 4).

**Fig. 5:**
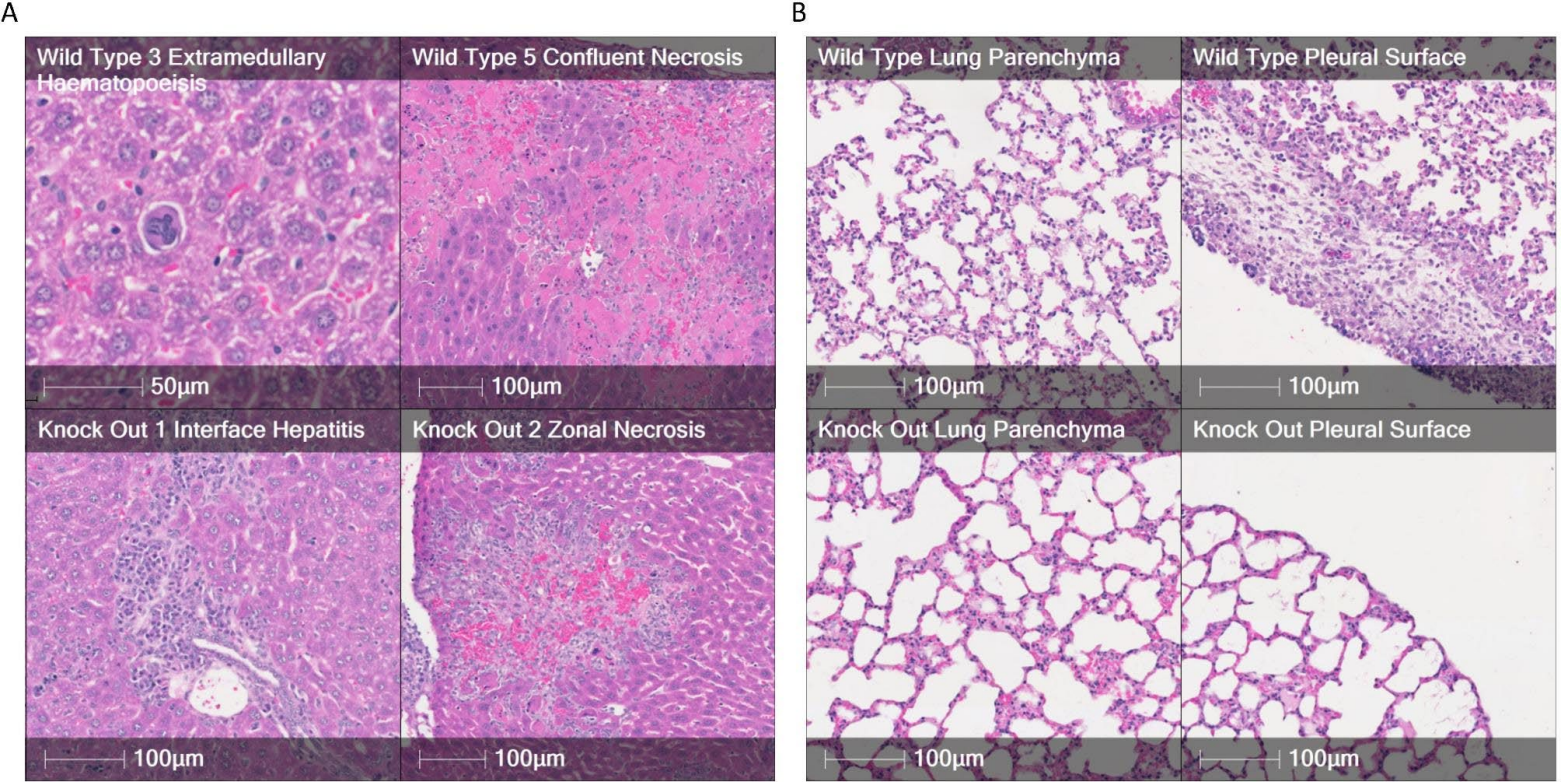
Photomicrographs of liver and lung tissues 5 weeks after injecting CHL-1 WT or TPC2 KO melanoma cell lines. **(A)** Liver tissues after injection with CHL-1 WT or TPC2 KO cells. **(B)** Lung tissue from mice injected with CHL-1 WT or TPC2 KO cells

Compared to the effect observed after the injection of TPC2 KO CHL-1 cells, the livers of mice injected with WT cells revealed a greater amount of extramedullary haematopoiesis, including megakaryocytes and erythroid precursors. The TPC2 KO-injected mouse livers demonstrated spotty and zonal necrosis, whereas the livers from WT-injected mice exhibited confluent and massive hepatic necrosis. There was evidence of interface hepatitis in the hepatic tissues of the TPC2 KO mice (Fig. 5A).

We also examined the lungs of mice injected with CHL-1 WT or TPC2 KO cells. Compared with the lungs of WT-injected mice, the lungs of TPC2 KO-injected mice exhibited increased numbers of neutrophils in the alveolar space and active chronic pleuritis with a proliferative response. The primary distinction between the TPC2 KO- and WT-injected mouse lungs was that the TPC2 KO-injected lungs exhibited a highly florid pleural response with acute and chronic inflammation (Fig. 5B).

### TPC2 KO cell-derived tumour growth resulted in changes in pro- and anti-inflammatory cytokine expression

**Fig. 6:**
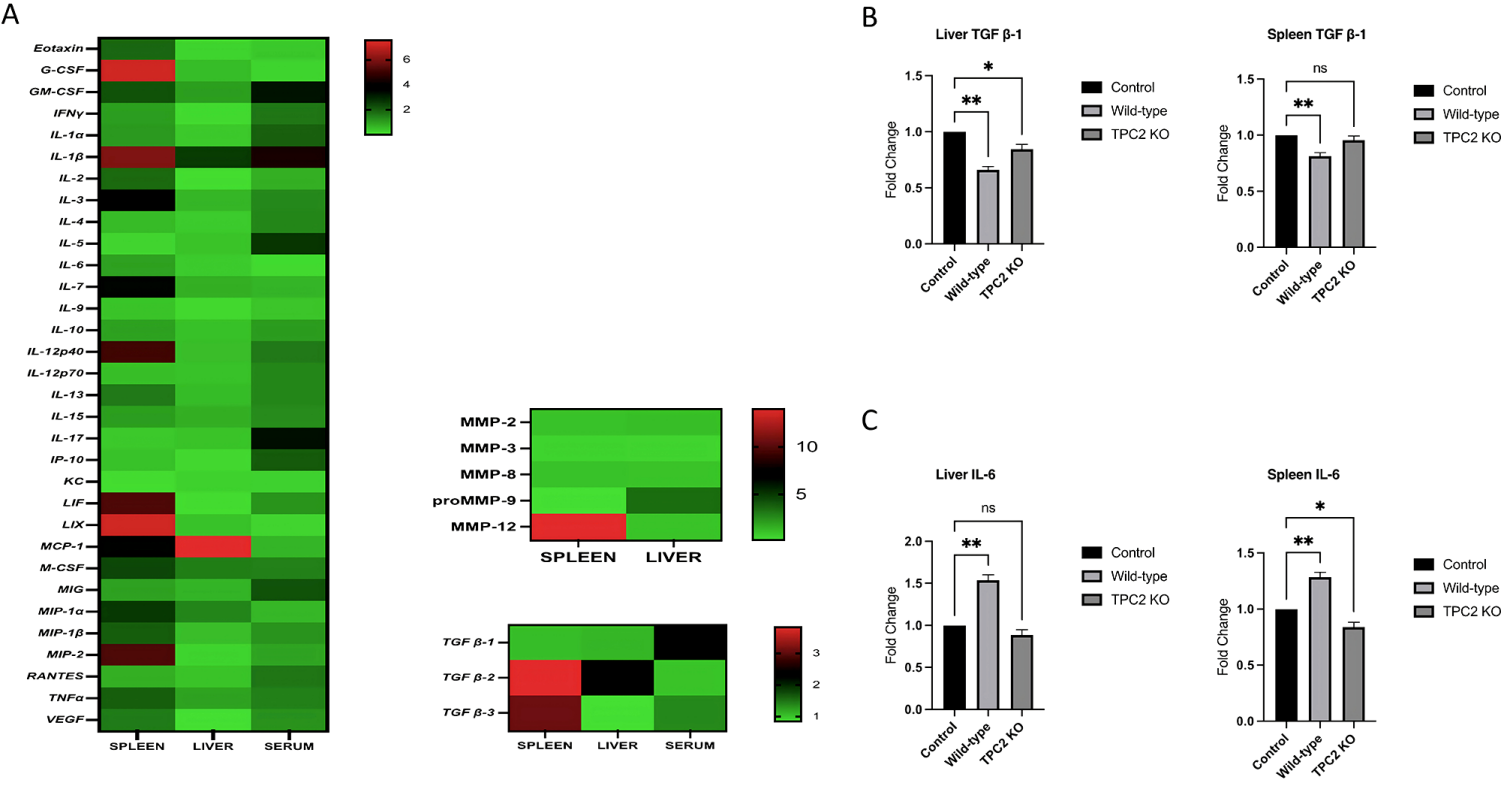
Cytokines, chemokines, and angiogenesis markers in the liver, spleen, and serum 5 weeks after injecting mice with CHL-1 WT or TPC2 KO melanoma cells. **(A)** Heatmap representing the overall number of differentially expressed markers in the spleen and liver tissues and serum after injection with CHL-1 WT or TPC2 KO cells. Data in heatmap represents the mean ± s.e.m. of three independent experiments. **(B)** qPCR validation of representative markers in spleen and liver tissues. Data in bar charts represent the mean ± s.e.m. of three independent experiments (* *p* < 0.05; ** *p* < 0.01; *** *p* < 0.001)

To identify the dysregulated factors that could be associated with the inflammatory response and tissue damage seen particularly in CHL-1 TPC2 KO-injected mice, we analysed 40 biomolecules consisting of cytokines, chemokines, and angiogenesis markers in the liver, spleen, and serum. The TPC2 KO-injected mouse samples differed significantly from the WT-injected mouse samples in approximately 72% of the analysed markers, as shown in the heat map in Fig. 6. Serum from TPC2 KO-injected mice showed substantially higher (> 2-fold) expression of GM-CSF, IL-1β, IL-5, IL-17, IP-10, MIG, and TGF β-1; the spleen showed a more than 2-fold change in G-CSF, GM-CSF, IL-1β, IL-3, IL-7, IL-12p40, LIF, LIX, MIP-2, TGF β-2, TGF β-3, and MMP-12; and the liver showed more than 2-fold upregulation in TGF β-2 and proMMP-9. In contrast, IL-6, KC, and LIX expression were significantly reduced in TPC2 KO injected mice serum compared to WT injected mice; the liver showed a reduction in Eotaxin, IL-1α, IL-2, IL-4, IL-6, IL-9, IL-10, KC, and LIF, while the spleen showed a significant reduction only in KC.

TGF-β, most commonly considered an anti-inflammatory cytokine, showed higher expression in all samples from TPC2 KO-injected mice. To further explore this finding, the level of TGF-β mRNA expression was assessed in liver and spleen tissues. Both tissues demonstrated significant upregulation in mice injected with TPC2 KO cells compared to mice injected with WT cells (Fig. 6B). Interestingly, compared to control mice, the livers and spleens of WT and TPC2 KO-injected mice showed lower TGF-β expression levels. Similarly, IL-6 levels were measured in the liver and spleen tissues and found to be considerably lower in the TPC2 KO group than those in the WT and control groups. Nonetheless, WT cells showed an increase compared to the control group (Fig. 6C).

## Discussion

Several recent studies have identified an important role for TPC2 in the progression of several different types of cancer [6, 10–12]. We recently demonstrated that when we generated a TPC2 KO in the human CHL-1 cell line, this had the opposite effect to TPC2 KO in MNT-1 human melanoma cell line, as it led to increased migratory and invasive behaviours [5]. These changes were caused by the activation of genes that are regulated by YAP/TAZ, which are critical regulators of tumourigenesis and metastasis. Our findings suggested that TPC2 KO may have different effects on cancer progression depending on the molecular and cellular context in which the loss of TPC2 function occurs, with potentially important clinical implications. However, one caveat of our previous study was that, like most studies that have investigated the role of TPC2 in cancer, the analysis was conducted solely using in vitro methods. To address this caveat, we studied the effect of TPC2 KO on B16 and CHL-1 cells injected into live mice.

As a preliminary to this in vivo analysis, we began by determining the real time proliferative rate of B16 and CHL-1 WT and TPC2 KO cells in vitro using the RTCA system. The process by which cancer cells replicate and divide their DNA into two stages is referred to as cell proliferation. Increased cell division suggests that the cancer is progressing more rapidly or becoming more aggressive. Therefore, it is important to study how the TPC2 KO affects this process. In real-time monitoring of cell proliferation, both CHL-1 and B16 TPC2 KO cells showed a similar pattern, with TPC2 KO cells initially exhibiting a greater cell index rate before the rate began to decline, whereas WT cells continued to show a high cell index. Increases in cell index should not always be interpreted as cell proliferation. Indeed, cell index values are dependent on the number of cells implanted in the wells, the manner in which the cells interact, the quality of interaction between the cells and the electrodes, and the overall morphology of the cells [13]. As we previously showed that CHL1 TPC2 KO cells didn’t show any significant change in proliferation rate or markers [5], the initial decline in cell index in both B16 and CHL-1 KO cells after around 100 h is likely to be due to morphological changes. Further analysis of TPC2 cell morphology is required in order to determine the role of TPC2 in this crucial characteristic.

Cancer cell lines are simple to cultivate in vitro, allow direct comparison of experimental data, and are frequently used to investigate the molecular principles underlying tumour cell biology. However, in vivo models better represent the complexity of tumourigenic and metastatic processes in organisms that cannot be replicated in vitro [14]. To analyze and compare the tumour progression between two models of melanoma (WT vs. TPC2 KO) and elucidate the role and the impact of the two-pore channel 2 (TPC2) gene in tumour growth on melanoma cells in vivo, we subcutaneously injected B16 and CHL-1 WT or TPC2 KO cells into mice. It was necessary to allow the tumour to reach that volume to achieve this comparative analysis. By doing so, we noticed a significant increase in tumour volume for the WT melanoma compared to the tumour volume of the TPC2 KO gene melanoma. This observation provided substantial evidence that the absence of the gene had a significant impact on slowing-down tumour growth which suggests that the presence of this gene might be essential for tumour growth and progression. Therefore, the comparative analysis between the two models played a crucial role in understanding the gene’s influence on tumour development.

We observed that WT cells developed significantly larger tumours than TPC2 KO cells in both cell lines. These in vivo findings may be explained by the in vitro findings mentioned above, in which TPC2 KO cells exhibited a severe reduction in cell growth rate after four days. Angiogenesis is another trait that could contribute to this outcome; it is the process through which new blood vessels develop from pre-existing vessels, allowing for tumour progression. As tumours grow subcutaneously, the formation of new blood vessels is crucial. TPC2 is a major regulator of the processes associated with the pathophysiology of neovascular age-related macular degeneration [15]. In addition, TPC2 functions in signalling pathways that promote angiogenesis triggered by VEGF [16]. Consequently, when tumours lack TPC2, this may lead to alterations in vasculogenesis around the tumour, which is essential for providing growth-promoting nutrients.

Despite the smaller size of TPC2 KO cell-derived tumours, they remarkably and unexpectedly produced greatly enhanced toxic effects on the whole organism. Thus, histopathological analysis of spleen tissues from mice injected with the TPC2 KO versions of both cell lines showed similar overall patterns, including lymphocyte atrophy, macrophage replacement, and suppurative and granulomatous inflammation. However, there were some differences between the two cell lines in terms of the histopathological changes observed. For example, in B16 TPC2 KO-injected mice, the spleen showed major pathological alterations, such as hyaline degeneration in the lymph nodes, a diffused appearance of the white pulp in the congested red pulp, and the presence of numerous large macrophages. In contrast, the spleens of CHL-1 TPC2 KO-injected mice showed a greater degree of suppurative and granulomatous inflammation than the spleens of B16 TPC2 KO-injected mice. Additionally, the spleens of the control and CHL-1 WT-injected mice lacked pale patches around the PALS and exhibited abundant lymphocytes, indicating lymphoid hyperplasia, which was not observed in the spleens of B16-injected mice. Therefore, although both the CHL-1 and B16 cell lines had similar effects on the spleen, there were differences in the extent and nature of the observed histopathological changes. These differences may be due to the unique properties of each cell line and their interactions with the mouse immune system.

The effects of tumours derived from B16 versus CHL-1 TPC2 KO cells on the liver showed significant differences. Injection of B16 TPC2 KO cells into mice led to severe alterations in the liver structure, such as fibrosis, massive infiltration of necrotic areas, congested veins, and cytoplasmic degeneration of hepatocytes. In contrast, injection of WT B16 cells resulted in a relatively healthy hepatic structure, but with small foci of inflammatory cells and the activation of Kupffer cells, the liver’s resident macrophages. These pathological characteristics were far less severe than those observed in the B16 TPC2 KO-injected group. In contrast, injection of TPC2 KO CHL-1 cells into mice led to spotty and zonal necrosis in the liver, whereas injection of WT CHL-1 cells resulted in confluent and massive hepatic necrosis. The livers of CHL-1 TPC2 KO-injected mice also demonstrated evidence of interface hepatitis, inflammation where liver tissue meets portal tracts, whereas those of WT-injected mice showed a greater amount of extramedullary haematopoiesis, blood cell formation outside the bone marrow, including megakaryocytes, cells that give rise to platelets, and erythroid precursors, immature cells becoming red blood cells. In addition, the lungs of mice injected with CHL-1 TPC2 KO cells exhibited increased numbers of neutrophils in the alveolar space and active chronic pleuritis with a proliferative response, which was not observed in the lungs of WT cell-injected mice.

Overall, our findings raise the question of how a smaller-sized TPC2 KO cell-derived tumour might cause far greater damage to organs in mice. One possibility is that the damage was due to an immune or inflammatory response. Immunosuppression is a hallmark of most cancer types and crucial for cancer development and progression [17, 18]. The body’s response to cancer is not unique and shares many similarities with inflammation and wound repair. Unresolved inflammation creates a microenvironment conducive to cellular remodelling and cancer cell development. The production of growth factors, cytokines, and chemokines is triggered by chronic tissue damage during healing. Cytokines and chemokines play vital roles in cancer-related inflammation, exerting direct and indirect effects on the proliferation and invasiveness of tumour cells. In recent years, studies have identified myeloid-derived suppressor cells (MDSC) as major contributors to the immunosuppressive tumour microenvironment (TME) [19]. Interestingly, these MDSC were found to accumulate in tumour-bearing animals, and animals with cancer accumulated MDSC in the liver [20] and spleen [21]. MDSC-induced liver damage was tumour-specific, as not all studied tumour models showed liver damage, despite the fact that MDSC proliferation was observed in all models [22]. Subcutaneous growth of B16 (melanoma) tumour cell lines on a C57BL/6 background results in moderate liver damage due to the accumulation of MDSC in the liver [22]. This evidence provides a rational explanation for the histopathological abnormalities observed following the injection of WT and TPC2 KO cells in comparison to the control group. However, this does not explain why organ damage was more pronounced in mice bearing TPC2 KO cell-derived tumours.

One possibility is that TPC2 KO cells may result in a markedly enhanced increase in MDSC accumulation in the liver and spleen. The accumulation of MDSC is a consequence of two processes: activation and expansion. MDSCs can be induced in vitro by various tumour-derived substances, including prostaglandin E2, interleukin-6, interleukin-10, interleukin-1, transforming growth factor (TGF), stem cell factor (SCF), and pro-angiogenic factors, including vascular endothelial growth factor (VEGF) [15, 16, 23, 24]. Interestingly, TPC2 KO-injection resulted in increased expression of several of these markers. Therefore, the loss of functional TPC2 may result in the overexpression of certain markers, resulting in an abnormal response in the host animal and the accumulation of excessive MDSC in organs. Alternatively, there may be an alternative mechanism underlying the more considerable damage to host organs associated with TPC2 KO tumours. Further investigation is required to identify the true underlying mechanism(s).

Another issue that remains to be resolved is the slightly different effects of TPC2 KO B16 and CHL-1 cell-derived tumours on the spleen and liver compared with the effects of their WT counterparts. To understand these differences, it is important to consider the origins of these two cell lines. The B16 line that we used, more specifically known as B16F0 to distinguish it from other B16 lines, is derived from an early-stage mouse melanoma that has been extensively studied in cancer research. It has been shown to express high levels of certain surface markers, such as CD44 and CD133, which are associated with cancer stem cells and may contribute to tumour initiation and resistance to chemotherapy. B16F0 cells also produce factors that promote angiogenesis, which is a key step in tumour growth and metastasis. Moreover, B16F0 cells have been reported to have the ability to evade immune surveillance by downregulating the expression of MHC class I molecules, which are essential for the recognition of tumour cells by cytotoxic T cells. In contrast, CHL-1 is a less well-known human melanoma cell line, and limited information is available about its specific properties. However, it has been shown to produce high levels of IL-8, a pro-inflammatory cytokine that has been implicated in tumour growth, invasion, and metastasis. B16 cells also express higher levels of genes related to angiogenesis and metastasis than CHL-1 cells [25]. Additionally, it has been shown that B16 cells are more resistant to natural killer cell-mediated cytotoxicity [26]. Therefore, the unique properties of each cell line, such as their ability to express specific surface markers, produce certain factors, and evade immune surveillance, may contribute to the observed differences in the effects of B16 and CHL-1 TPC2 KO cell-derived tumours in the liver and spleen.

In summary, our in vivo analysis revealed that, for both B16 and CHL-1 cell lines, WT cells generated a substantially larger tumour than TPC2 KO cells. Remarkably, histopathological investigations revealed that B16 and CHL-1 TPC2 KO cell-derived tumours were associated with significant degradation of liver and spleen tissues relative to WT cell-derived tumours, and that CHL-1 TPC2 KO cell-derived tumours were also associated with the degradation of lung tissue. To explain this unexpected finding and based on the analysis of the expression of pro- and anti-inflammatory cytokines in different tumour-bearing mice, we suggest that there may be an enhanced response by MDSC in mice with TPC2 KO tumours. Further studies are required to confirm this finding or identify an alternative underlying mechanism. Overall, our findings emphasise the importance of combining in vitro and in vivo investigations when elucidating the role of TPC2 in cancer.

## Data Availability

The datasets during and/or analysed during the current study are available from the corresponding author on reasonable request.
